# Results of surgical treatment of necrotizing soft tissue infections. A 15-years retrospective cohort study of a single center of emergency surgery

**DOI:** 10.1007/s00423-026-04038-x

**Published:** 2026-03-31

**Authors:** Silvia Tedesco, Marta Di Grezia, Gaia Altieri, Caterina Cina, Valeria Fico, Filomena Misuriello, Giada Bracalente, Edoardo Piras, Giuseppe Brisinda

**Affiliations:** 1 Emergency Surgery and Trauma Center, Fondazione Policlinico Universitario A Gemelli, IRCCS, Rome, 00168 Italy; 2 Thoracic Surgery, Fondazione Policlinico Universitario A Gemelli, IRCCS, Rome, 00168 Italy; 3 Catholic School of Medicine “Agostino Gemelli”, Rome, 00168 Italy; 4 Department of Medical and Surgical Sciences, Fondazione Policlinico Universitario A Gemelli, IRCCS, Largo Agostino Gemelli 8, Rome, 00168 Italy

**Keywords:** Debridement, Humans, Necrosis, Soft Tissue Infections, Risk factors, Surgery

## Abstract

**Background:**

Although rare, necrotizing soft tissue infections carries a high risk of mortality and morbidity. Extensive surgical debridement of necrotic tissues until healthy tissue is the cornerstone of management. Despite this, in-hospital mortality remains high for these patients.

**Methods:**

We evaluated the experience of an Emergency Surgery center over a 15-year period. We evaluated factors related to in-hospital mortality. Secondary outcomes were assessment of morbidity, need for intensive care admission, risk of amputation, and outcomes related to the timing of surgery.

**Results:**

One hundred and eight patients were included in this retrospective cohort study. All patients, except one (99.1%), underwent surgical debridement. Of these, 64 patients (59.3%) required multiple revisions (2.0 ± 2.7). Negative pressure wound therapy was applied to 52 patients (48.1%), for a mean duration of 21.7 ± 23.3 days. The mortality rate was 25.9% (28 patients). Ninety patients (83.3%) developed a complication during hospitalization, most of which were severe (20 patients grade III and 64 grade IV). Patients operated on within 10 h experienced a reduction in the rate of major complications (*P* = 0.02). Fourteen patients (13%) underwent limb amputation and 37 patients (34.3%) presented motor deficits at discharge.

**Conclusions:**

Necrotizing soft tissue infections are a challenge for the emergency surgeon. An accurate diagnosis is not always possible and rapid intervention improves the results. Currently, there are no elements that allow a definitive diagnosis and a determining factor for a correct and accurate diagnostic framework is the competence of the surgeon.

## Introduction

Necrotizing soft tissue infections (NSTIs) are severe infections characterized by necrosis in the soft tissues of the body [1–4]. These infections typically involve the fascia, subcutaneous tissue, and sometimes muscle. The term NSTIs covers a range of infections that can occur in different anatomical locations and develop after the integrity of the skin (or mucosa) has been breached. Although rare, NSTIs carries a high risk of mortality and morbidity, expressed in terms of sequelae, the risk of amputation, and complications arising from prolonged stays in intensive care unit (ICU) and hospital settings [5, 6].

Over the decades, interest in this condition has led the scientific community to establish its correct definition. However, when analyzing the therapeutic proposals and their effects on surgical outcomes, there has not been a reduction in associated mortality over the past few decades. Although, from a purely surgical standpoint, the therapeutic options — such as extensive necrosectomy, drainage of purulent collections, or fasciotomies — are technically straightforward to perform, the fundamental pitfall of this condition remains the diagnosis [7–9]. Currently, there are no laboratory or radiological elements that provide a definitive diagnosis. Evidence suggests that, although certain scoring systems and typical clinical signs can provide a strong diagnostic suspicion, the only way to make a diagnosis (whether microbiological, histological, or clinical) is through the surgical procedure itself [10, 11]. It is a challenging task for the surgeon to identify patients potentially affected by this condition to determine the appropriate surgical indication with the aim of minimizing the incidence of major complications and mortality [12].

In this retrospective cohort study we evaluated the experience of our Emergency Surgery Unit in the surgical treatment of patients affected by NSTIs. The objectives of the study were to identify factors related to in-hospital mortality, the onset of complications and late sequelae, the risk of amputation, and the most appropriate surgical timing to improve patient outcomes.

## Materials and methods

This single-center, retrospective cohort study was conducted on patients followed up between January 2009 and June 2024 at Fondazione Policlinico Universitario Agostino Gemelli IRCCS, Rome, Italy. All patients discharged with a diagnosis of “necrotizing fasciitis” were collected. This study follows the STROBE reporting guidelines [13]. The study was conducted in accordance with the Declaration of Helsinki. All patients provided written consent before the surgical procedures.

Due to the lack of specific coding for NSTIs, we only included patients whose hospital discharge records contained the ICD code 728.86 (ICD-9-CM). We excluded pediatric patients (age < 18 years), pregnant women, and patients who, despite the coding of necrotizing fasciitis, did not exhibit signs of necrotizing infection during surgical exploration. Additionally, patients with incorrect coding on their hospital discharge records were excluded from the analysis.

The evaluated parameters included age at time of diagnosis, sex, comorbidities, preceding trauma or surgery, laboratory findings, signs of shock at admission in emergency department (ED), radiological finding at admission, surgical strategy and number of surgical revisions, antibiotic therapy, postoperative wound care, need of ICU and ICU length of stay (ICU-LOS), decreased physical functions, complications, global length of stay (G-LOS).

Comorbidities diagnosed by primary care physicians or other specialty physicians, as documented in the medical records, were considered. We grouped them as diabetes, heart disease, obesity, malignancy, chronic renal insufficiency, chronic liver disease, immunosuppression, peripheral arterial disease, intravenous drug use, and chronic alcohol use. To evaluate comorbidities, the Charlson Comorbidity Index (CCI) score [14] was calculated for each patient. Systemic severity at inclusion was measured using the American Society for of Anesthesiologists (ASA) [15] physical status classification, the Shock Index [16], the Simplified Acute Physiology Score (SAPS) II [17], the Sequential Organ Failure Assessment (SOFA) score [18], and the Acute Physiologic Assessment and Chronic Health Evaluation (APACHE) II score [19]. The Laboratory Risk Indicator for Necrotizing Fasciitis (LRINEC) score [20] was calculated based on preoperative measurements. Clavien-Dindo classification [21] was used to determine the severity of postoperative complications. According to classification of NSTIs [22], microbiological findings were identified as polymicrobial infection (Type I), monomicrobial infection (Type II), Vibrio spp. infection (Type III) and fungal infection (Type IV).

Subgroups were identified in relation to age (< 65 years and ≥ 65years). LRINEC cut off ≥ 6 was established for NSTIs and patients were then classified into 3 risk groups based on their point score: Low (< 6 points), Intermediate (6–7 points) and High (≥ 8 points) risk. We divided the patients into two cohorts: those admitted to the ICU and those who either did not require admission or were admitted for only one day. Other subgroups were identified in relation to the type of infection (type I polymicrobial and type II monomicrobial), and to the timing of surgery. In this last case, we performed a double evaluation, the first identifying the surgical timing cut off as 10 h (Surgery < 10 h and Surgery > 10 h) and in the second case using the cut off as 12 h (Surgery < 12 h and Surgery > 12 h) from admission to the ED.

### Aim of the study

The primary outcome of this study was in-hospital mortality. Factors related to in-hospital mortality were investigated. Secondary end points were the evaluation of factors that influenced morbidity (Clavien-Dindo grade III and IV), the need for ICU admission, risk of amputation, type of infection (mono- or poly-microbial) and outcomes related to the timing of surgery.

### Statistical analysis

All data were analyzed using the Statistical Package for Social Science version 30.0 (IBM Corp., Armonk, NY, USA). Categorical variables are presented as percentages. Continuous data are reported as mean ± standard deviation (± SD) or median and interquartile range as appropriate. Due to repeated measures within subjects, a random-effects ANOVA was employed to correct for the systematic error. Categorical data were analyzed using the χ^2^ or Fisher exact test. Continuous data were compared using the T-test or the Mann–Whitney U test. The presence of a normal distribution was assessed using the Shapiro-Wilk test. A multivariate logistic regression was performed by constructing models that took into consideration the potential factors influencing the course of the disease that in the univariate analysis had a p value < 0.25, according to the Hosmer–Lemeshow rule. A P value of less than 0.05 was considered statistically significant.

## Results

Demographic data of the 108 patients enrolled in our study are shown in Table 1. The mean age was 60.8 ± 14.9 years with 38.9% of patients aged 65 years or older. Seventeen patients developed NSTI after surgical procedures (gynecologic, abdominal and orthopedic surgery). In 8 cases (7.4%) a superficial soft tissue infection has evolved into the necrotizing form. In only one case (0.9%) no specific cause of NSTI has been identified. Nine patients (8.3%) had no comorbidity. NSTIs can affect various anatomical sites. The principal locations included the head and neck, trunk and extremities.

**Table 1:** Demographic data

Parameters	Data	%
Mean age (years)	60.8 ± 14.9	-
Age < 65 y	66	61.1
Age ≥ 65 y	42	38.9
Sex		
Male	64	59.3
Female	44	40.7
BMI	27.5 ± 8.3	-
Comorbidities		
Diabetes	46	42.6
Peripheral artery disease	27	25.0
Cardiomyopathy	13	12.0
Liver disease	11	10.2
Chronic kidney disease	19	17.6
Immunosuppression	16	14.8
Malignancy	14	13.0
Smoke	30	27.8
Chronic alcohol disease	8	7.4
Drug addiction	5	4.6
Obesity	54	50.0
Etiology NSTI		
Trauma	12	11.1
Diabetes	22	20.4
Recent surgery	17	15.7
Immunosuppression	6	5.6
Odontogenic	6	5.6
Skin breaches		
- Insect bite	3	2.8
- Injection drug use	3	2.8
- Ulcer and small abscesses	8	7.4
Secondary to		
- Intestinal perforation	10	9.3
- Enteric fistula	6	5.6
- Arterial thrombosis	2	1.9
Anatomical site		
Head and neck	17	15.7
Trunk	16	14.8
Extremities		
Upper limb and arms	13	12.0
Lower limb	44	40.7
Pelvis	2	1.9
Perineum and genitalia (Fournier’s gangrene)	13	12.0
Multifocal	5	4.6
Skin sign		
Swelling	101	93.5
Ecchymosis	34	31.5
Bullae	16	14.8
Pus	35	32.4
Necrosis	32	29.6

Categorical variables are presented as percentages. Continuous data are reported as mean ± SD or median and interquartile range as appropriate

The mean CCI score was 3.9 ± 2.7, and the majority of the patients had an ASA score of 3 (63 patients, 58.3% - Table 2). A correlation between mean age and ASA score was documented (ASA I 29.7 ± 5.0 years, ASA II 54.6 ± 12.3 years and ASA III 66.7 ± 12.8 years, *P* < 0.00001). In the ED, 32 patients (29.9%) exhibited signs of shock. Mean LRINEC score was 7.9 ± 2.3, and most of the patients (58.3%) were in the high-risk group (score ≥ 8). Out of the 108 patients, only 78 were admitted to ICU, where the SAPS II scores were calculated within the first 24 h. Clinical findings were observed in 94.4% of patients, while 5.6% exhibited no skin signs at admission due to NSTI. Nearly all patients (100 patients, 92.6%) underwent pre-operative contrast-enhanced computed tomography (CECT). Only one patient (0.9%) had an ultrasound study, while 7 patients (6.5%) did not undergo any imaging studies before surgery. Analysis of the CECT scans revealed edema (86.9%), subcutaneous gas (72%), abscess formation (76.6%), and necrosis (0.9%).

**Table 2:** Vital parameters, laboratory tests and gravity scores at admission in ED

Variables	
Vital Parameters	
SBP (mmHg)	120.0 ± 24.7
DPB (mmHg)	70.4 ± 16.5
HR (bpm)	98.9 ± 20.0
SpO2 (%)	95.1 ± 5.4
Temperature (°C)	37.2 ± 0.9
GCS	13.4 ± 3.2
Laboratory Tests	
Hgb (g/dl)	11.4 ± 2.5
WBC (10^9/l)	16.7 ± 10.5
HCT (%)	33.5 ± 7.2
Creatinine (mg/dl)	1.6 ± 1.3
CRP (mg/l)	233.3 ± 117.0
Procalcitonin (ng/ml)	14.6 ± 35.9
LAC	2.7 ± 2.0
pH	7.4 ± 0.1
**Scores**	
ASA 1	4 (3.7%)
ASA 2	41 (38.0%)
ASA 3	63 (58.3%)
Shock Index	0.9 ± 0.3
CCI	3.9 ± 2.7
SOFA	4.2 ± 4.0
qSOFA	0.8 ± 1.2
APACHE II	14.7 ± 8.0
SAPS II*	36.6 ± 18.0
LRINEC score	7.9 ± 2.3
Low risk (< 6)	15 (13.9%)
Intermediate risk (6–7)	30 (27.8%)
High risk (≥ 8)	63 (58.3%)

* SAPS II data are calculated on 78 patients. Categorical variables are presented as percentages. Continuous data are reported as mean ± SD or median and interquartile range as appropriate. *SBP *Systolic blood pressure, *DBP* diastolic blood pressure, *HR* heart rate, *SpO2* peripheral capillary oxygen, *GCS* Glasgow coma scale, *Hgb* hemoglobin, *WBC* white blood cells; *HCT* hematocrit, *CRP* C-reactive protein, *LAC* Lactate level. *SI* Shock index, *SOFA* Sequential Organ Failure Assessment, *qSOFA* Quick Sequential Organ Failure Assessment, *APACHE II* Acute Physiology and Chronic Health Disease Classification System, *SAPS II* Simplified Acute Physiology Score

All patients presenting with signs of NSTIs were commenced on empirical, broad-spectrum antibiotics covering gram negative, gram positive, and anaerobic organisms until microbiology cultures allowed for rationalization, as per institutional health-board guidelines. This included an anti-toxin, such as clindamycin, if there was suspicion of NSTIs or evidence of septic shock. The average time of the antibiotic therapy, considering the first broad-spectrum antibiotic regimen and possible escalation or de-escalation, was 39.4 ± 29.7 days.

All patients in the study, except one (99.1%), underwent surgery. In most cases, the procedure performed was skin and subcutaneous tissue debridement with fasciotomy of the affected muscular aponeurosis. Of these, 64 patients (59.3%) required multiple revisions (mean = 2.0 ± 2.7). In the first 24 to 48 h following surgical source control, negative pressure wound therapy (NPWT) was applied in 52 patients (48.1%). The average duration of NPWT application was 21.7 ± 23.3 days.

A polymicrobial infection (Type I) has been identified in 60 patients (55.6%), a monomicrobial infection (Type II) in 29 (26.9%) and a fungal infection (Type IV) in 2 patients (1.9%). No type III infection was identified. In 15.7% of cases, no pathogens were detected from the microbiological samples (Table 3). Staphylococcus Aureus was identified in six patients (20.7%), with two classified as methicillin-resistant (MRSA).

**Table 3:** Microbiological findings

Microorganism	Type I	Type II
Enterococci	24 (39.0%)	1 (3.4%)
- E. Avium	2 (3.3%)	
- E. Faecalis	12 (20.0%)	1 (3.4%)
- E. Faecium	7 (11.7%)	
- E. Hirae	1 (1.7%)	
- E. Raffinosus	2 (3.3%)	
Bacteroides spp.	**18 (29.9%)**	**0**
- B. Vulgatus	2 (3.3%)	
- B. Fragilis	12 (20.0%)	
- B. Ovatus	2 (3.3%)	
- B. Thetaiotaomicron	2 (3.3%)	
Escherichia Coli	**22 (36.3%)**	**4 (13.8%)**
Clostridioides	**14 (20.1%)**	**0**
- C. Innocuum	2 (3.3%)	
- C. Clostridiforme	3 (5.0%)	
- C. Bifermentas	1 (1.7%)	
- C. Ramosum	4 (6.7%)	
- C. Sporogenes	2 (3.4%)	
Streptococci	**21 (24.9%)**	**10 (34.5%)**
- S. Pyogenes	2 (3.3%)	7 (24.1%)
- S. Agalactiae	3 (5.0%)	
- S. Constellatus	9 (15.0%)	2 (6.9%)
- S. Anginosus	5 (8.3%)	1 (3.4%)
- S. Gallolyticus	2 (3.3%)	
Pseuodomonas	**5 (8.4%)**	**0**
- P. Aeruginosa	4 (6.7%)	
- P. Putida	1 (1.7%)	
Proteus spp.	**10 (16.7%)**	**1 (3.4%)**
- P. Vulgaris	1 (1.7%)	
- P. Mirabilis	9 (15.0%)	1 (3.4%)
Acinetobacter baumannii	**6 (10.0%)**	**2 (6.9%)**
Staphylococci	**10 (16.8%)**	**6 (20.7%)**
- S. Aureus	7 (11.7%)	6 (20.7%)
- S. Lugdunensis	1 (1.7%)	
- S. Haemolyticus	1 (1.7%)	
- S. Simulans	1 (1.7%)	
Klebsiella spp.	**9 (15.0%)**	**2 (6.9%)**
- K. Oxytoca	2 (3.3%)	
- K. Pneumoniae	7 (11.7%)	2 (6.9%)
Others		
- Actinomyces	2 (3.3%)	**1 (3.4%)**
- Aerococcus spp	1 (1.7%)	
- Citrobacter freundii	2 (3.3%)	
- Eggerthella lenta	2 (3.3%)	
- Fusobacterium	2 (3.3%)	
- Aeromonas	1 (1.7%)	
- Morganella morganii	4 (6.7%)	**1 (3.4%)**
- Corinebacterium striatum	3 (5.0%)	
- Fingoldia magna		**1 (3.4%)**

In all patients, the length of ICU stay and hospital stay were 8.9 ± 11.1 days and 46.4 ± 25.4 days, respectively.

### Mortality

The mortality rate was 25.9% (28 patients). The factors associated with mortality are reported in Table 4. A significant correlation was found with uraemia (*P* = 0.001), creatinine (*P* = 0.04), procalcitonin (*P* = 0.001), platelets (*P* = 0.009), base excess (*P* = 0.004) and lactate (*P* = 0.02). All severity scores, except the Shock Index, are correlated with mortality. Analyzing the radiological elements (subcutaneous gas, abscess formation, edema, necrosis) and the various locations of the infection, no statistically significant correlation with mortality was found. As regard surgical treatment, no significant correlation with mortality was found. The need for ICU admission (65% in survivors vs. 92.8% in deceased, *P* = 0.005) was found to be significant correlated with mortality. Only type II infections was found to be significant correlated with mortality. The length of hospital stay was 21.7 ± 13.1 days in deceased patients and 41.0 ± 26.6 days in surviving patients (*P* = 0.0005).The need of vasoactive agents (OR 8.352, 95% CI 1.869–37.306, *P* = 0.005) and the presence of necrosis as a clinical finding (OR 8.184, 95% CI 1.617–41.41, *P* = 0.01) resulted in association with mortality at the multivariate analysis.

**Table 4:** Parameters associated with in-hospital mortality

Variables	Survivor	Deceased	*P* values
Age	57.3 ± 14.0	71.7 ± 12.6	0.0001
M/F	48/32	15/13	0.2
ASA			
- ASA1	4 (5%)	0 (0%)	0.01
- ASA2	36 (45%)	5 (17.8%)	
- ASA3	40 (50%)	23 (82.1%)	
BMI	28.2 ± 9.3	25.5 ± 3.9	0.1
CCI	3 (2–4)	6 (4–8)	0.001
Diabetes	33 (41.2%)	13 (46.4%)	0.6
Peripheral artery disease	16 (20%)	11 (39.3%)	0.04
Cardiomyopathy	7 (8.7%)	6 (21.4%)	0.07
Liver disease	8 (10%)	3 (10.7%)	0.9
Chronic kidney disease	8 (10%)	11 (39.3%)	0.001
Immunosuppression	9 (11.2)	6 (21.4%)	0.1
Malignancy	9 (11.2%)	5 (17.8%)	0.3
Smoke	25 (31.2%)	5 (17.8%)	0.1
Chronic alcohol disease	6 (7.5%)	1 (3.5%)	0.4
Drug addiction	4 (5%)	0 (0%)	0.2
ED LOS (hours)	6.2 ± 5.4	6.0 ± 5.4	0.8
Signs of shock	20 (25.0%)	13 (46.4%)	0.03
Skin’s signs	72 (90%)	24 (85.7%)	0.5
- Swelling	71 (88.7%)	25 (89.3%)	0.9
- Ecchymosis	25 (31.3%)	10 (35.7%)	0.6
- Bullae	10 (12.5%)	6 (21.4%)	0.2
- Pus	29 (36.3%)	6 (21.4%)	0.1
- Necrosis	18 (22.5%)	12 (42.8%)	0.03
SBP (mmHg)	121.1 ± 24.6	117.1 ± 24.6	0.4
DBP (mmHg)	72.1 ± 15.9	65.8 ± 17.4	0.08
HR (bpm)	99.1 ± 20.1	98.6 ± 20.2	0.9
qSOFA	0.8 ± 1.2	1.0 ± 1.3	0.4
Temperature (°C)	37.1 ± 1.0	37.2 ± 1.1	0.8
GCS	15 (14–15)	15 (10–15)	0.08
Need of vasoactive agents	24 (30%)	22 (78.5%)	0.001
CRRT	9 (11.2%)	16 (57.1%)	0.001
Severity scores			
Shock Index	0.8 ± 0.3	0.8 ± 0.3	0.7
SOFA	3.5 ± 3.8	6.6 ± 3.7	0.0003
Apache II score	13.1 ± 6.5	19.7 ± 10.0	0.0001
SAPS score	32.2 ± 14.5	50.4 ± 20.9	0.0001
LRINEC score	7.4 ± 2.4	8.7 ± 2.6	0.02

Categorical variables are presented as percentages. Continuous data are reported as mean ± SD or median and interquartile range as appropriate. *ICU* Intensive care unit, *LOS ICU* Intensive care unit length of stay, *NPWT* Negative pressure wound therapy, *ATB* Antibiotic therapy, *ED LOS* Emergency department length of stay, *SBP* Systolic blood pressure, *DBP* Diastolic blood pressure, *HR* Heart rate, *GCS* Glasgow Coma Scale, *CRRT* Continuous renal replacement therapy, *BMI* Body Mass Index, *CCI* Charlson Comorbidity Index

### Morbidity

Ninety patients (83.3%) developed a complication during the hospitalization. Four patients (3.7%) presented a grade I complication, 31 patients (28.7%) had grade II complication, 20 patients (18.5%) developed a grade III and 64 patients (59.4%) had a grade IV complication. The variables associated with the development of major complications (Clavien-Dindo grade III and IV) were drug use (17.8% in Clavien-Dindo III-IV vs. 0% in Clavien-Dindo I-II, *P* = 0.002), chronic alcohol disease (17.8% in Clavien-Dindo III-IV vs. 3.8% in Clavien-Dindo I-II, *P* = 0.03), and malignancies (21.4% in Clavien-Dindo III-IV vs. 5.8% in Clavien-Dindo I-II, *P* = 0.03). PCT values were significantly higher (4.0 ng/ml) in patients with Clavien-Dindo III-IV complications compared to those with Clavien-Dindo I-II complications (1.5 ng/ml, *P* = 0.01). At multivariate analysis, factors associated with severe morbidity (Clavien-Dindo III and IV) are the presence of malignancy (OR 13.50, 95% CI 2.05–88.77, *P* = 0.007) and value of procalcitonin (OR 1.06, 95% CI 1.02–1.11, *P* = 0.006).

### Necessity of ICU

Seventy-seven patients were admitted to the ICU for 12.3 ± 11.5 days. Diabetes emerged as the only cause of infection with a statistically significant difference (*P* = 0.008 – Table 5). Heart rate (*P* = 0.01) and procalcitonin levels (*P* < 0.001) showed an association with a length of stay in ICU > 1 day. Among the severity scores, only APACHE II and SAPS showed significant differences between the two groups. Additionally, different results were observed for the duration of antibiotic therapy. At multivariate, factor associated with ICU admission were NSTIs at lower limb (OR 0.31, 95% CI 0.11–0.88, *P* = 0.02), procalcitonin levels (OR 1.06, 95% CI 1.00-1.12, *P* = 0.02) and antibiotic therapy duration (OR 1.04, 95% CI 1.01–1.07, *P* = 0.002).

**Table 5:** Variables associated with ICU admission

Variables	ICU > 1 day	ICU ≤ 1 day	*P*
Age	61.6 ± 15.0	58.6 ± 14.9	0.3
BMI (kg/m2)	25.7 (20.8–66.8)	24.6 (18.7–70.3)	0.4
Diabetes (cause)	8 (12.1%)	14 (33.3%)	0.008
Site of infection			
- Head and neck	14 (21.2%)	3 (7.1%)	0.05
- Trunk	13 (19.7%)	3 (7.1%)	0.07
- Upper limbs and arm	7 (10.6%)	6 (14.3%)	0.5
- Lower limb	20 (30.3%)	24 (57.1%)	0.006
- Pelvis	1 (1.5%)	1 (2.4%)	0.7
- Fournier’s gangrene	7 (10.6%)	6 (14.3%)	0.5
- Multifocal	4 (6.1%)	1 (2.4%)	0.3
CCI	3.8 ± 2.8	3.9 ± 2.6	0.8
SBP (mmHg)	116.8 ± 26.3	125.2 ± 21.4	0.08
DPB (mmHg)	69.2 ± 17.2	72.4 ± 15.4	0.3
HR (bpm)	102.6 ± 20.9	93.2 ± 17.4	0.01
Temperature (°C)	37.1 ± 1.0	37.3 ± 0.9	0.2
CRP (mg/l)	239.1 ± 119.3	224.3 ± 114.3	0.5
PCT (ng/ml)	5.27 (0-300)	1.42 (0–47)	< 0.001
WBC (10^9/l)	16.0 ± 10.9	17.7 ± 9.9	0.4
LDH (mU/ml)	312.3 ± 154.6	300.6 ± 175.9	0.7
CK (UI/l)	124 (15-6066)	74 (9-4815)	0.08
pH	7.4 ± 0.1	7.4 ± 0.1	0.6
BE	-2.6 ± 6.1	-0.9 ± 5.0	0.1
Lactate	2.9 ± 2.0	2.6 ± 1.9	0.4
SOFA	5.4 ± 4.0	1.5 ± 2.1	< 0.001
qSOFA	0.9 ± 1.2	0.6 ± 1.1	0.2
APACHE II	16.1 ± 7.9	12.7 ± 7.7	0.03
SAPS	39.5 ± 17.2	31.8 ± 18.2	0.02
Shock Index	0.9 ± 0.3	0.7 ± 0.2	0.05
LRINEC	8.0 ± 2.5	7.2 ± 2.3	0.1
ATB therapy duration	47.8 ± 60.6	26.2 ± 18.5	0.008
N° of surgical revisions	2.1 ± 2.8	1.7 ± 2.3	0.4

Categorical variables are presented as percentages. Continuous data are reported as mean ± SD or median and interquartile range as appropriate. *BMI* Body Mass Index, *CCI* Charlson Comorbidity index, *SBP* Systolic blood pressure, *DBP* Diastolic blood pressure, *HR* Heart rate, *CRP* C-reactive protein, *PCT* Procalcitonin, *WBC* White blood cells, *LDH* Lactate dehydrogenase, *CK* Creatinine kinase, *ATB* Antibiotic therapy

### Risk of amputation

Fourteen patients (13%) underwent limb amputation. Of these patients, 3 (2.8%) underwent upper limb amputation (either a portion such as amputation up to the elbow or complete amputation with shoulder disarticulation), and 11 patients (10.2%) underwent lower limb amputation (amputation of the leg or thigh). In 9 patients, due to extensive loss of skin and subcutaneous tissue, reconstructive plastic surgery was necessary to achieve not only the best aesthetic outcome but also functional recovery. In these patients, a single or multiple dermo-epidermal graft was used in 5 patients; in two patients, it was necessary to perform a pedunculate flap to achieve the best result and two patients underwent the procedure of INTEGRA application.

Diabetes (76.9% in amputation group vs. 37.2% in no-amputation group, *P* = 0.01) and peripheral artery disease (69.2% in amputation group vs. 23.2% in no-amputation group, *P* = 0.002) resulted associated with the need of amputation. A LRINEC score > 8 demonstrated a statistically significant association with the risk of amputation (92.3% in amputation group vs. 13.9% in no-amputation group, *P* = 0.03). The mean LRINEC score was higher in patients who underwent surgical amputation (9.4 ± 1.9 vs. 7.8 ± 1.9, *P* = 0.01). Patients who underwent amputation had a higher mortality rate, likely due to the severity of their condition (46.1% vs. 16.3%, *P* = 0.02). With optimal source control achieved through amputation, the overall length of stay was shorter for these patients (20.1 ± 13.5 days vs. 41.2 ± 24.5 days, *P* < 0.001). At multivariate analysis, diabetes (OR 10.32, 95% CI 1.68–63.26, *P* = 0.01) resulted associated at the risk of amputation.

### Polymicrobial vs. monomicrobial infection

Immunosuppression and chronic liver disease are risk factors for monomicrobial infections (Table 6). The etiology most strongly associated with the development of monomicrobial infections included trauma (*P* = 0.007) and insect bites (*P* = 0.04). In contrast, the causes linked to polymicrobial infections were intestinal perforations (*P* = 0.02) and progression from abscesses (*P* = 0.02). Factors associated with monomicrobial infection in the multivariate analysis were chronic liver disease (OR 0.18, 95% CI 0.36–0.92, *P* = 0.03) and traumatic etiology (OR 0.91, 95% CI 0.19–0.43, *P* = 0.003).

**Table 6:** Variables associated with polymicrobial (type I) vs. monomicrobial (type II) NSTIs

Variables	Type I	Type II	*P*
Age ≥ 65 years	23 (38.3%)	10 (34.5%)	0.7
Comorbidities			
- diabetes	29 (48.3%)	10 (34.5%)	0.2
- peripheral artery disease	9 (15%)	8 (27.6%)	0.1
- drug addiction	4 (6.7%)	1 (3.4%)	0.5
- immunosuppression	6 (10%)	8 (27.6%)	0.03
- chronic liver disease	3 (5%)	6 (20.7%)	0.02
- chronic alcohol abuse	4 (6.7%)	4 (13.8%)	0.2
- smoke	16 (26.7%)	10 (34.5%)	0.4
- chronic kidney disease	7 (11.7%)	8 (27.6%)	0.06
- malignancy	10 (16.7%)	2 (6.9%)	0.2
- cardiomyopathy	7 (11.7%)	1 (3.4%)	0.2
- obesity	34 (56.7%)	10 (34.5%)	0.05
Causes			
- trauma	3 (5%)	7 (24.1%)	0.007
- diabetes	13 (21.7%)	3 (10.3%)	0.1
- drug injection	3 (5%)	0 (0%)	0.2
- previous surgery	8 (13.3%)	6 (20.7%)	0.3
- immunosuppression	2 (3.3%)	4 (13.8%)	0.06
- insect bites	0 (0%)	2 (6.9%)	0.04
- odontogenic origin	2 (3.3%)	3 (10.3%)	0.1
- intestinal perforation	9 (15%)	0 (0%)	0.02
- intestinal fistula	5 (8.3%)	1 (3.4%)	0.3
- artery thrombosis	1 (1.7%)	0 (0%)	0.4
- abscess	10 (16.7%)	0 (0%)	0.02
- skin breach	4 (6.7%)	2 (6.9%)	0.9
Localization			
- head and neck	8 (13.3%)	5 (17.2%)	0.6
- trunk	10 (16.7%)	3 (10.3%)	0.4
- Upper limbs and arm	4 (6.7%)	5 (17.2%)	0.1
- Lower limb	26 (43.3%)	11 (37.9%)	0.6
- Pelvis	1 (1.7%)	1 (3.4%)	0.5
- Fournier’s gangrene	11 (18.3%)	2 (6.9%)	0.1
- Multifocal	2 (3.3%)	2 (6.9%)	0.4

Categorical variables are presented as percentages. Continuous data are reported as mean ± SD or median and interquartile range as appropriate

### Timing of surgery

When comparing groups of patients who underwent surgery within 10 h of arriving at the ED to those operated on after this timeframe (Table 7), we found that patients operated on within 10 h experienced a reduction in the rate of major complications and in the duration of antibiotic therapy. By analyzing data from patients operated on within 12 h of arrival at the ED and those who underwent surgery after 12 h, we found that surgical intervention performed within 12 h is associated with a significant increase in the need of use of vasoactive agents and the necessity for NPWT (Table 8). At multivariate analysis, the only factor associated with surgery performed within 12 h was the need of vasoactive agents (OR 3.33, 95% CI 1.03–10.71, *P* = 0.04).

**Table 7:** Variables associated with surgery < 10 h

Variables	Surgery < 10 h	Surgery > 10 h	*P*
Age	57.5 ± 15.2	61.4 ± 14.9	0.2
BMI	26.8 ± 4.9	27.7 ± 8.9	0.6
qSOFA	0.9 ± 1.2	0.8 ± 1.2	0.5
Shock Index	1.0 ± 0.5	0.8 ± 0.2	0.04
SOFA	5.1 ± 4.9	3.9 ± 3.7	0.01
APACHE II	17.1 ± 10.2	14.0 ± 7.3	0.04
SAPS	40.2 ± 26.7	35.7 ± 15.4	0.002
LRINEC	7.4 ± 2.8	7.8 ± 2.5	0.5
IOT	6 (30%)	39 (44.8%)	0.3
Need of vasoactive agents	7 (35%)	39 (44.8%)	0.4
CRRT	2 (10%)	23 (26.4%)	0.1
Need of ICU	14 (70%)	64 (73.5%)	0.8
Need of surgical revisions	13 (65%)	51 (58.6%)	0.5
NPWT	13 (65%)	39 (44.8%)	0.09
Complications			
- CD grade I	1 (5%)	3 (3.4%)	0.7
- CD grade II	5 (25%)	26 (29.8%)	0.7
- CD grade III	0 (0%)	20 (22.9%)	0.02
- CD grade IV	1 (5%)	7 (8.0%)	1.0
- CD grade III plus grade IV	1 (5%)	27 (31.0%)	0.02
Mortality	6 (30%)	22 (25.3%)	0.7
Motor deficits	3 (15%)	34 (39.0%)	0.06
Amputation	2 (10%)	12 (13.8%)	1.0
Need of plastic surgery	0 (0%)	9 (10.3%)	0.1
NPWT duration	22.2 ± 23.8	23.2 ± 23.6	0.8
ATB therapy duration	31.5 ± 23.7	42.4 ± 55.2	0.0001
LOS ICU	10.1 ± 11.5	8.5 ± 11.1	0.5
LOS	29.6 ± 20.8	39.4 ± 26.9	0.07

Categorical variables are presented as percentages. Continuous data are reported as mean ± SD or median and interquartile range as appropriate. *IOT* Orotracheal intubation, *CRRT* Continuous renal replacement therapy, *ICU* Intensive care unit, *CD* Clavien-Dindo, *LOS ICU* Intensive care unit length of stay, *ATB* Antibiotic therapy, *LOS* global length of stay

**Table 8:** Variables associated with surgery < 12 h

Variables	Surgery < 12 h	Surgery > 12 h	*P*
Age	59.7 ± 14.8	61.1 ± 15.1	0.6
BMI	27.9 ± 8.1	27.3 ± 8.5	0.7
qSOFA	0.9 ± 1.1	0.8 ± 1.2	0.6
Shock Index	0.9 ± 0.4	0.7 ± 0.8	0.02
SOFA	4.3 ± 4.3	4.1 ± 3.8	0.7
APACHE II	15.8 ± 8.6	14.0 ± 7.5	0.2
SAPS	37.3 ± 22.7	36.0 ± 15.0	0.7
LRINEC	7.3 ± 3.0	8.0 ± 2,2	0.1
IOT	21 (55.3%)	23 (33.3%)	0.03
Need of vasoactive agents	21 (55.3%)	24 (34.8%)	0.04
CRRT	10 (26.3%)	14 (20.3%)	0.4
Need of ICU stay	31 (81.6%)	48 (69.6%)	0.1
Surgical revision	24 (63.1%)	40 (58%)	0.6
Need for NPWT	24 (63.1%)	27 (39.1%)	0.02
Complications			
- CD grade I	1 (2.6%)	3 (4.3%)	0.6
- CD grade II	10 (26.3%)	21 (30.4%)	0.6
- CD grade III	8 (21%)	12 (17.4%)	0.6
- CD grade IV	3 (7.9%)	5 (7.2%)	0.9
- CD grade III plus grade IV	11 (28.9%)	17 (24.6%)	0.6
Mortality	9 (23.7%)	18 (26.1%)	0.7
Motor deficit	12 (31.6%)	25 (36.2%)	0.6
Amputation	3 (7.9%)	11 (15.9%)	0.3
Need for plastic surgery	4 (10.5%)	5 (7.2%)	0.5
NPWT duration	29.7 ± 31.8	19.1 ± 18.0	0.1
ATB therapy duration	43.8 ± 76.4	38.2 ± 26.5	0.5
LOS ICU	10.6 ± 13.1	7.8 ± 9.8	0.2
LOS	36.3 ± 31.5	36.5 ± 21.8	0.9

Categorical variables are presented as percentages. Continuous data are reported as mean ± SD or median and interquartile range as appropriate. *IOT* Orotracheal intubation, *CRRT* Continuous renal replacement therapy, *ICU* Intensive care unit, *CD* Clavien-Dindo, *LOS ICU* Intensive care unit length of stay, *ATB* Antibiotic therapy, *LOS* global length of stay

### Long-term sequelae

At discharge, 37 patients (34.3%) exhibited motor deficits (e.g., walking with aids, the need for prosthetic devices, difficulties with the joints of the upper limbs, etc.). Furthermore, 69 patients (63.9%) returned home, 2 patients (1.9%) were transferred to another hospital, 8 (7.4%) were admitted to a rehabilitation facility, and 2 (1.9%) to a nursing home. Among 80 surviving patients, only 4 of them (3.7%) were readmitted to the hospital within 30 days.

## Discussion

NSTIs are severe conditions characterized by rapid progression and extensive tissue destruction [6], often requiring intensive care management. Although epidemiological data remain limited, the clinical burden is substantial, with a high proportion of patients requiring ICU admission due to systemic involvement and comorbidities [23–25]. The average age of patients with NSTIs is between 50 and 60 years, with a slight predominance of males [26, 27]. In the present study, a mean age of 60.8 ± 14.9 years and a predominance of male patients with severe clinical conditions are confirmed. A significant number of patients required prolonged ICU stays, reflecting the severity of illness and complexity of postoperative care.

Mortality rates for NSTIs remain high, typically ranging between 20% and 30% [28, 29], in line with the 28.5% observed in our study. Disease severity plays a central role in predicting outcomes, as demonstrated by the association between higher severity scores (APACHE II, SAPS, SOFA) and mortality. Clinical parameters such as hypotension and vasopressor requirement have been strongly associated with worse outcomes in prior studies [30–33], emphasizing the importance of early hemodynamic assessment. In our analysis, skin necrosis at ED admission emerged as an independent predictor of mortality. Additionally, laboratory markers—including urea, creatinine, procalcitonin, platelet count, base excess, and lactate—were significantly associated with mortality, highlighting their potential role in early risk stratification.

Early diagnosis of NSTIs remains challenging. Initial clinical signs are often nonspecific, and neither laboratory nor imaging findings are consistently reliable in the early phase. In our cohort, procalcitonin was the only laboratory parameter significantly associated with both major complications and prolonged ICU admission. While severity scores correlate with outcomes, they lack specificity for NSTIs. The LRINEC score appears to be correlated with the risk of amputation (9.4 ± 1.9 in patients undergoing amputation vs. 7.8 ± 1.9 in non-amputated patients, *P* = 0.01; High risk group 92.3% in amputated vs. 13.9% in non-amputated, *P* = 0.03) and the severity of the clinical picture. However, we have noted that this score cannot be used as an absolute diagnostic tool for NSTIs, as 15 patients (13.9%) with a diagnosis of NSTIs had an LRINEC < 6.

Given these diagnostic limitations, prompt surgical intervention remains the cornerstone of management. Early and extensive surgical debridement is essential not only for source control but also for diagnostic confirmation [34–39]. Evidence suggests that early intervention significantly improves outcomes [40], with delays beyond 12 h associated with increased mortality and complications. More recent guidelines recommend, in suspected NSTIs, performing surgical debridement within 6 h. This approach would have benefits on mortality compared to surgical treatment delayed beyond 6 hours [29, 41]. Surgery delayed beyond 12 h is associated with the need for more frequent surgical re-explorations to locally control infection and a higher incidence of septic shock [42]. In our study, although early surgery (within 10 h) did not significantly reduce mortality—likely due to sample size—it was associated with a lower rate of major complications (Clavien-Dindo III-IV, *P* = 0.02).

Current guidelines recommend surgical re-exploration every 12 to 24 h after the initial debridement [34]. It is common for patients to require 2 to 4 debridement to obtain an optimal source control [35]. The importance of repeated debridement is confirmed by the results of a study in which exploration scheduled at 24 h compared to later ones (48 h) is associated with a lower mortality (7% vs. 33%) and a lower acute kidney injury (9% vs. 33%) [43]. In the current study, 59.3% of patients required multiple revisions (mean = 2.0 ± 2.7) and NPWT was used in 48.1% of patients, for a mean duration of 21.7 ± 23.3 days. In this context, there is no consensus on the appropriateness of NPWT, and the optimal timing to start NPWT remains unclear. NPWT has recently been shown to reduce mortality when compared to conventional dressings [44] and it may be more effective in patients with aerobic or polymicrobial NSTIs [45]. In small series of patients, NPWT allows for the closure of complex wounds [46, 47].

The rates of major complications, including amputation and motor deficits, were consistent with existing literature. The amputation rate in our study (13%) falls within the wide range reported (7–53%) [48, 49, 50, 51], while the incidence of motor deficits reflects the long-term functional impact of NSTIs [52, 53].

Misdiagnosis remains a critical issue, particularly in the early stages of disease when cutaneous manifestations may be absent [54]. This contributes to delays in diagnosis and treatment, negatively affecting outcomes [55]. Our findings reinforce the importance of maintaining a high index of suspicion and prioritizing clinical judgment. Patients with NSTIs typically present with signs of severe infection, which sometimes precede skin lesions [56]. In cases of diagnostic uncertainty, early surgical exploration is often justified to avoid treatment delays. Imaging modalities may support diagnosis but should not postpone surgical intervention in critically ill patients.

A multidisciplinary approach is essential for optimal management, involving surgeons, intensivists, and infectious disease specialists [40, 57, 58]. Indeed, 72.2 of our patients required admission to ICU, primarily due to the severity of the condition with systemic involvement, but also because many elements of cohort presented multiple comorbidities. Some of these were significant risk factors for the development of the infection itself, with specific correlations for the type of infection, polymicrobial or monomicrobial. Although debridement and surgical source control are central to the diagnosis and treatment of NSTIs, adequate antibiotic therapy—initially empirical and broad-spectrum—followed by targeted therapy based on microbiological isolations, is a fundamental prerequisite for favorable clinical outcomes. Most patients received empirical antibiotic therapy, sometimes starting from their arrival in the ED [59]. However, this was not always tailored to a diagnostic suspicion of NSTIs, and therefore not always initiated according to the recommended regimen: a carbapenem with the addition of clindamycin in case of suspected GAS infection, an anti-MRSA drug (vancomycin, linezolid, or daptomycin), and an aminoglycoside in cases of shock, which was then de-escalated based on microbiological results. The early initiation of antibiotic therapy may also explain the occasional presence of negative microbiological samples [60–62]. The relative incidence of type I and type II NSTIs varies considerably [2, 63]. Usually, type I is much more common in immunocompromised patients or in elderly subjects with multiple comorbidities [64], unlike type II which is observed in subjects of all ages and without immune compromise [56]. In the present study, immunosuppression (27.6% vs. 6%, *P* = 0.03), liver disease (20.7% vs. 5%, *P* = 0.02), and chronic kidney failure (27.6% vs. 11.7%, *P* = 0.06) appear to be correlated with the onset of monomicrobial infections.

### Limitations

This study has several limitations. First, its retrospective design introduces potential selection and information biases, including incomplete data collection and variability in clinical documentation. Second, the single-center setting may limit the generalizability of the findings to other institutions with different patient populations, resources, or clinical practices. Third, the sample size, particularly for subgroup analyses, may have reduced the statistical power to detect significant associations, such as the impact of early surgical intervention on mortality. Additionally, heterogeneity in treatment approaches—including timing of surgery, antibiotic regimens, and use of NPWT—may have influenced outcomes.

## Conclusion

The wide range of clinical presentations of NSTIs — from initial localized expression to systemic involvement leading to septic shock — is well represented in our cohort. This not only reflects the clinical variability of the condition but also encompasses a 15-year period of our diagnostic and therapeutic improvement, with enhanced diagnostic algorithms and more prompt, decisive interventions. It is necessary to overcome the conceptual limitations that assign diagnosis to the physician and curative treatment to the surgeon, through the application of techniques and procedures that are more or less complex. Currently, there are no elements that allow for a definitive diagnosis; thus, the key factor in accurate diagnostic framing is the surgeon’s expertise. Given the type of clinical presentation and the timing of symptom development, the first medical-patient interaction regarding this condition often occurs with an abscess in the ED. For this reason, the professional most likely to develop empirical diagnostic skills is the acute care surgeon.

## Data Availability

No datasets were generated or analysed during the current study.

## Declarations

### Statement on Generative AI and AI-Assisted Technologies in the Writing Process

During the preparation of this work, the authors did not use any AI tools to improve the language and readability of the manuscript.
